# A Capacitive Micromachined Ultrasonic Transducer-Based Resonant Sensor Array for Portable Volatile Organic Compound Detection with Wireless Systems

**DOI:** 10.3390/s19061401

**Published:** 2019-03-21

**Authors:** Inug Yoon, Gayoung Eom, Sungwoo Lee, Bo Kyeong Kim, Sang Kyung Kim, Hyunjoo J. Lee

**Affiliations:** 1 School of Electrical Engineering, Korea Advanced Institute of Science and Technology (KAIST), Daejeon 34141, Korea; inug.yoon@kaist.ac.kr (I.Y.); gayoung.eom@kaist.ac.kr (G.E.); lsw9403@kaist.ac.kr (S.L.); 2 Information & Electronics Research Institute, Korea Advanced Institute of Science and Technology (KAIST), Daejeon 34141, Korea; 3 Molecular Recognition Research Center, Korea Institute of Science and Technology (KIST), Seoul 02792, Korea; bk0357@khu.ac.kr (B.K.K.); sangk@kist.re.kr (S.K.K.)

**Keywords:** resonant gas sensor, multiplex detection, VOC, CMUT, selectivity

## Abstract

The development of portable volatile organic compound (VOC) sensors is essential for home healthcare and workplace safety because VOCs are environmental pollutants that may critically affect human health. Here, we report a compact and portable sensor platform based on a capacitive micromachined ultrasonic transducer (CMUT) array offering multiplex detection of various VOCs (toluene, acetone, ethanol, and methanol) using a single read-out system. Three CMUT resonant devices were functionalized with three different layers: (1) phenyl-selective peptide, (2) colloids of single-walled nanotubes and peptide, and (3) poly(styrene-co-allyl alcohol). As each device exhibited different sensitivities to the four VOCs, we performed principal component analysis to achieve selective detection of all four gases. For the simultaneous detection of VOCs using CMUT sensors, the changes in the resonant frequencies of three devices were monitored in real time, but using only a single oscillator through an electrically controlled relay to achieve compactness. In addition, by devising a wireless system, measurement results were transmitted to a smartphone to monitor the concentration of VOCs. We used multiple sensors to obtain a larger number of fingerprints for pattern recognition to enhance selectivity but interfaced these sensors with a single read-out circuit to minimize the footprint of the overall system. The compact CMUT-based sensor array based on a multiplex detection scheme is a promising sensor platform for portable VOC monitoring.

## 1. Introduction

Volatile organic compounds (VOCs) are widely used chemical ingredients in industrial processes and household products [1], but the inhalation of VOCs causes nervous system disorders, asthma, and cancers. Thus, accurate monitoring of VOCs in the workplace and at home is essential [2]. Currently, the standard analytical methods to determine the levels of VOCs are mass spectrometry (MS) and gas chromatography (GC)-MS [3]. These methods exhibit high sensitivity and selectivity to a target chemical in a gas mixture but are often expensive and require bulky instruments. To overcome the limitations of conventional analytical techniques, various miniaturized resonant chemical sensors for the detection of VOCs have been proposed, including surface acoustic wave (SAW), cantilever, quartz crystal microbalance (QCM), and capacitive micromachined ultrasonic transducer (CMUT) sensors [4,5,6,7]. These approaches have successfully addressed the issue of achieving high sensitivity while miniaturizing the sensor, which is essential for portable applications [8,9]. In addition, unlike metal-oxide based miniaturized sensors that operate at an elevated temperature, resonant chemical sensors operate at ambient temperature.

However, despite its importance, achieving selectivity is still a challenge. There are three main approaches employed to achieve selectivity for a miniaturized chemical sensor: (1) development of a highly sensitive detection layer, (2) use of a long microfluidic column to separate the gases from a mixture, and (3) use of an array of identical sensors to perform pattern recognition. For instance, various chemically sensitive layers to hazardous gases, such as formaldehyde (HCHO), carbon monoxide (CO), and nitrogen dioxide (NO_2_), have been reported, but these layers are still responsive to changes in the environment such as temperature and humidity [10,11,12]. In addition, it is inevitable for a chemically sensitive layer to exhibit cross-sensitivity to other chemicals. An alternative method to overcome this inherent cross-sensitivity is to exploit redundancy from a large dataset obtained from an array of sensors [13,14]. If reliable fingerprints could be extracted from the sensor outputs, performing pattern recognition on a large dataset would enhance the estimation accuracy of chemical concentrations. However, the use of a large array of sensors requires an equal number of read-out circuits. Thus, despite the small size of the sensor array, the overall system that includes the read-out circuits and power sources increases in size in order to accommodate multiple sensors (or channels) [15,16]. Therefore, it is still a challenge to achieve a sensor that is selective, yet portable.

**Figure 1:**
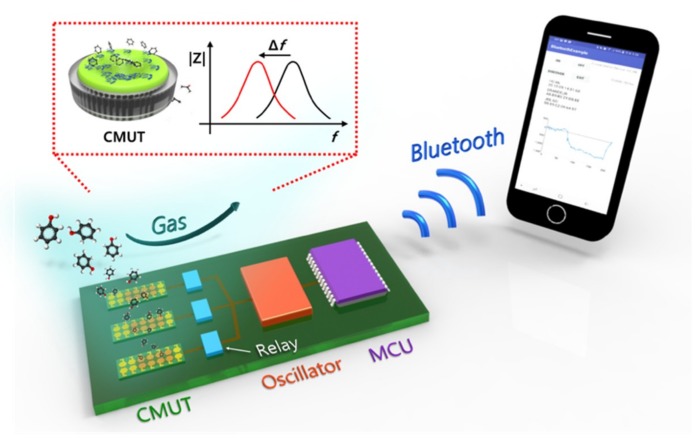
Conceptual schematic diagram of a wireless, portable VOC sensor system based on multiple CMUT resonant sensors and multiplex detection scheme with an actively controlled relay.

Thus, in this work, we aimed to develop a portable sensor system based on multiple sensors to achieve selectivity but with only a single read-out circuit to achieve compactness. Specifically, we implemented a CMUT-based portable VOC sensor system with a multiplex detection scheme where three CMUT sensors were interfaced with a single oscillator circuit through an electrically controlled relay (Figure 1). 

CMUT, which is a MEMS-based resonator that detects chemicals through the mass-loading mechanism, is suitable for the miniaturized bio/chemical sensor systems due to its small size, high sensitivity, and array structure [11]. For the selective detection of VOCs, three CMUT devices were coated with different chemically sensitive layers: (1) phenyl-selective peptide, (2) a colloid of single-walled carbon nanotubes and phenyl-selective peptide (SWCNT/peptide), and (3) poly- (styrene-co-allyl alcohol) (PSAA). In the proposed simultaneous multiplex detection scheme, the output frequencies of three CMUT sensors were sequentially sampled every 3 s and transmitted the data through the Bluetooth antenna to a smartphone in real time. The different response patterns exhibited by these sensors to four VOCs (toluene, acetone, ethanol, and methanol) were analyzed through a principal component analysis (PCA) to estimate chemical concentrations with enhanced accuracy. Because of the excellent scalability in terms of the number of channels, the proposed CMUT-based chemical sensor system is a promising portable platform for selective detection through massive parallelism.

## 2. Materials and Methods

The proposed portable VOC sensor system with wireless transmission consists of an array of resonant sensors, chemically sensitive layers to functionalize the resonant sensors, and a multiplex read-out system. In this section, design consideration and implementation of each component are discussed in detail followed by a description of experimental chemical setup to evaluate the sensor performance.

### 2.1. Design and Fabrication of CMUT Sensors

**Figure 2:**
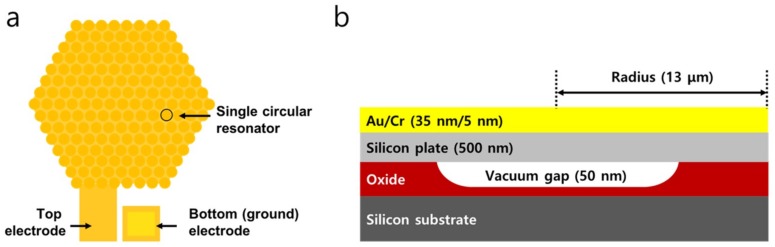
(**a**) Schematic diagram of a CMUT sensor composed of multiple circular resonators connected in parallel. For electrostatic actuation, DC and AC voltages are applied across the top and bottom electrodes. (**b**) Schematic diagram of a cross-section of a single circular resonator.

A single CMUT sensor consists of 331 identical circular resonators with a radius of 13 μm (Figure 2a). The individual circular resonator is structured as a capacitor which consists of two electrodes supported by an oxide post (Figure 2b). The top resonating plate is composed of a composite plate of a 500-nm-thick silicon plate and a 40-nm-thick metal layer, which is separated by a 50-nm-thick vacuum cavity (Figure 2b). This vacuum cavity reduces the viscous damping on the one side of the resonant plate and thus enhances the quality factor. The highly-doped silicon substrate acts as the bottom electrode. There is a thin insulation layer on top of the bottom electrode to prevent shortage between the top and bottom electrodes during operation. The capacitive structure is actuated electrostatically by applying DC and AC voltage across the two electrodes. The resonant frequency of a 26-μm-wide, 540-nm-thick circular resonator is approximately 10.4 MHz. 331 of these circular resonators are placed in parallel in a hexagonal structure with shared top and bottom electrodes to form a single CMUT device (Figure 2a). When a mass of the top resonating plate changes, the resonant frequency also changes. This mass sensor is converted to a chemical sensor by placing a chemically-sensitive layer on top of the resonating plate. When a chemical is absorbed on the layer, the mass of the resonant structure increases and thus, the resonant frequency decreases (i.e., through mass-loading mechanism). By measuring the shift in the resonant frequency, we estimate the concentration of the absorbed chemical.

CMUTs were fabricated based on a previously reported wafer-bonding method but with a different layout [17]. The fabrication process started with thermal oxidation (900 °C, wet O_2_) of a highly doped silicon wafer which was used as the bottom electrode. A 50-nm-thick oxide layer was grown through this oxidation process. The silicon dioxide layer was then patterned and etched which defined the cavity of CMUT. After etching the oxide layer in an etchant, second thermal oxidation (900 °C, wet O_2_) was performed to form the 80-nm-thick insulation layer on the bottom electrode to prevent electrical shortage between the two electrodes. Next, the oxidized and patterned wafer was bonded to a silicon-on-insulator (SOI) wafer through fusion wafer-bonding in a vacuum chamber. Thus, the gap was vacuum encapsulated. The substrate of the SOI wafer was then removed by CMP followed by wet etching (5% TMAH). After removing the substrate, the buried oxide (BOX) layer was removed in a 6:1 buffered oxide etch (BOE) solution. Next, the top silicon layer of the SOI wafer was patterned in a hexagonal shape to define the single CMUT device through dry etching. After dry etching, the oxide layer was patterned to expose the bottom silicon. Finally, a gold/chromium (Au/Cr) layer was deposited and patterned to form contact pads for both the bottom and the top electrodes of the CMUT device.

### 2.2. Chemically Sensitive Layers

Since the CMUT resonant sensor is a sensitive mass sensor but with less selectivity to VOCs, we coated the surface of the gold layer on the top plate with various chemically sensitive layers: phenyl- selective peptide (peptide layer), colloids of SWCNT and phenyl-selective peptide (SWCNT/peptide layer), and PSAA. For the peptide layer, a phenyl-selective peptide with a DNPIQAVP sequence (Peptron, Daejeon, South Korea) was used, which showed high binding affinity to toluene [18]. Peptide solution (0.4 μL, 100 μM) was dropped on CMUT devices and incubated in the homemade humanized chamber for 5 h. Peptides were attached through Au-thiol bond. For the SWCNT/peptide layer, the solution was prepared by mixing 40 μL of SWCNT (1 mg/mL, Sigma-Aldrich, St. Louis, MO, USA) and 60 μL of the peptide (100 μM) in a microtube followed by sonication for dispersion. 0.2 μL of the mixture was dropped on the device and spin-coated with 2000 rpm for 60 s. For the PSAA layer, 0.15 μL of 0.5 wt% PSAA was dropped on CMUT devices and dried in an ambient environment. These coating protocols set the thickness of each layer to be approximately 50 nm to minimize the loading on the 540-nm-thick resonant plate.

### 2.3. Design and Implementation of Wireless, Portable Sensor Platform

**Figure 3:**
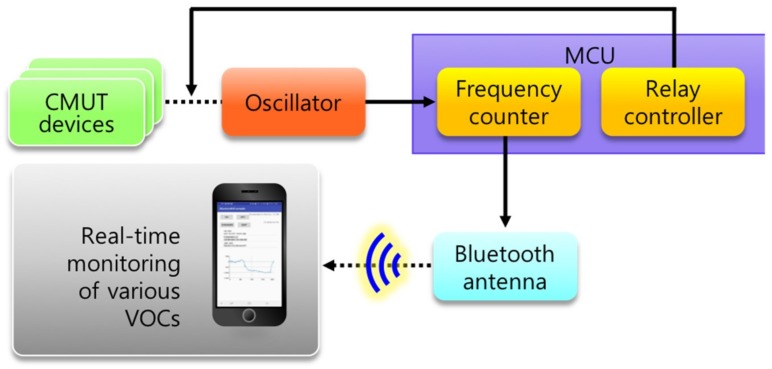
Block diagram of sensor interface scheme for the wireless, portable CMUT sensors.

The proposed portable VOC sensor interface consists of an oscillator, microcontroller unit (MCU), and a Bluetooth antenna for wireless transmission (Figure 3). The first block is an oscillator that tracks the shifts in the resonant frequencies of the CMUT sensors in real time. We chose Colpitts topology which tracks the parallel resonance because CMUT resonators exhibited a higher quality factor (Q) at the parallel resonance [15,19]. 

**Figure 4:**
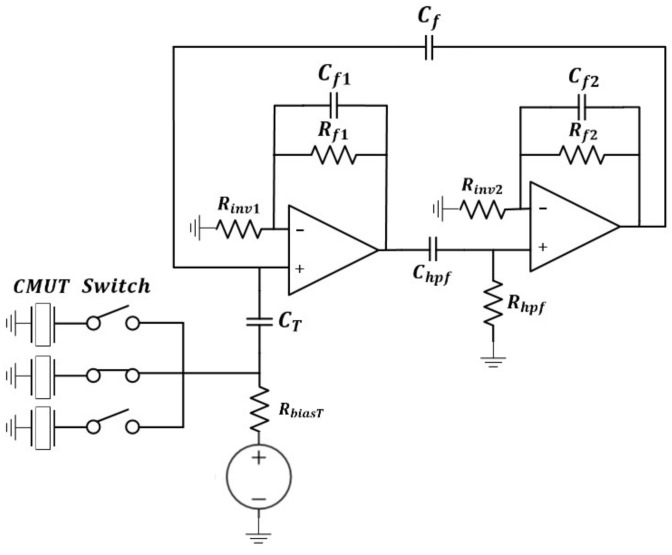
Schematic diagram of the Colpitts oscillator interfaced with three CMUT devices through an actively controlled relay.

The values of electrical components were selected through a publicly available circuit simulation tool (LTspice, Linear Technology, Milpitas, CA, USA). Prior to designing the circuit parameters, the input impedance (amplitude and phase) of the CMUT device was measured using an impedance analyzer (E4990A, Keysight Technologies, Santa Rosa, CA, USA) and was fitted to an equivalent circuit model. Using this equivalent circuit model, an oscillator circuit of Colpitts topology was designed and implemented on a custom printed circuit board (PCB) using discrete circuit components (Figure 4). Specifically, two closed-loop operational amplifier stages (R_f1_, C_f1_, R_f2_, C_f2_) and a high-pass filter (R_hpf_, C_hpf_) were connected in series to form a bandpass filter. The output of the second stage amplifier was capacitively divided through two capacitors (C_f_, C_T_) to limit the peak-to-peak signal amplitude and was connected to the first amplifier forming a global feedback loop.

For practical implementation, the DC bias voltage for the CMUT sensors was supplied by a voltage source through a bias-T. Also, to provide interconnection to the oscillator circuit, the contact pads of the CMUT device were wire-bonded to the PCB pads using gold wires. Prior to the implementation of the portable platform, the output frequency of the oscillator was measured using a high-resolution frequency counter (SR620, SRS, Sunnyvale, CA, USA). The sensor responses of three functionalized CMUT sensors were first evaluated. Because each device was coated with a different chemically sensitive layer, each device exhibited its own unique fingerprint (e.g., fall and rise time and frequency shift) to four VOCs (i.e., toluene, methanol, ethanol, and acetone). Thus, we applied these unique fingerprints as inputs to principal component analysis (PCA) to classify an input gas into four VOCs.

Next, MCU (dsPIC30F3013, Microchips, Chandler, AZ, USA) was programmed to: (1) control relay, (2) count frequency, and (3) establish Bluetooth communication. To achieve compactness, the outputs of three CMUT devices were connected to a single oscillator through an actively controlled relay (Figure 4). Only one CMUT device was electrically connected to the oscillator at a given time. The relay was electrically controlled by the relay controller in MCU and switched between three oscillators every 3 s. Specifically, three output pins of the MCU was programmed to be ‘high’ for 3 s in a 9-s period in a sequential manner. The frequency of the input signal was counted using two internal timers of MCU. While the first timer triggered an interrupt signal every 1 s, another timer counted the number of the rising edge of the oscillation signal during the 1-s interrupt. The total count over the 1-s period was outputted as the oscillation frequency. The Bluetooth communication function was implemented to build the connection between the read-out board and the Android smartphone. The measured frequencies from CMUT devices were transmitted to a smartphone through a Bluetooth antenna. Finally, a custom-designed graphic user interface (GUI) in the smartphone displayed the concentration of VOCs which was estimated by analyzing the frequency change. The portable board was powered using a 9 V battery and a voltage regulator circuit (LM7805, Texas Instruments, Dallas, TX, USA). 9 V was down converted to 5 V using a CMOS voltage converter (ICL7660, Texas Instruments, Dallas, TX, USA).

### 2.4. Experimental Setup for Detection of VOCs

We used the mass flow controlled evaporation system to generate different concentrations of VOCs: ethanol (Samchun Chemicals, Daejeon, Korea), acetone (Samchun Chemicals, Daejeon, Korea), methanol (Alfa Aesar, Tewksbury, MA, USA), and toluene (Alfa Aesar, Tewksbury, MA, USA). In specific, two mass flow controllers (MFC, IMC1300, ISVT, Gyeonggi-do, Korea) and a bubbler were used with nitrogen as a carrier gas. The carrier gas was passed through a VOC liquid in a bubbler to generate a saturated VOC vapor at the output of the bubbler. This saturated VOC vapor was then merged with the carrier gas. We adjusted the concentration of the final VOC vapor fixed at 2000 sccm for all experiments. Different concentrations of VOCs were passed over the CMUT sensors through an acrylic chamber (11 × 4 × 4 mm) through a tubing. All experiments were performed at a constant laboratory temperature of 24 °C.

## 3. Results and Discussion

### 3.1. Characterization of CMUT Sensors

**Figure 5:**
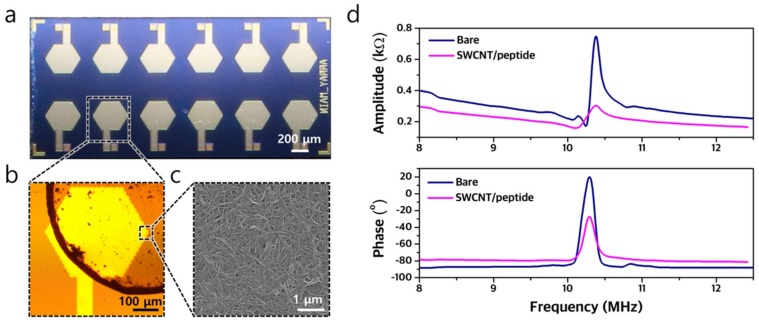
(**a**) Optical image of the fabricated array of CMUT devices. (**b**) Optical image of a single CMUT device coated with a 50-nm-thick SWCNT/peptide layer. (**c**) SEM image of SWCNT/peptide layer coated on top of the CMUT sensor. (**d**) Plot of input impedance (amplitude and phase) of the CMUT device biased at 6.2 V before and after the coating of SWCNT/peptide.

We successfully fabricated a 2.5 mm × 5 mm array that consists of 12 identical 10.4-MHz CMUT devices (Figure 5a). These sensors were then coated with three distinctive chemically sensitive layers (Figure 5b). The scanning electron microscope (SEM, Nova 230 instrument) image of SWCNT/peptide layer coated on a CMUT device clearly showed the successful deposition (Figure 5c). The input impedance of resonant devices was measured before and after coating to confirm that additional layer did not dampen the resonant characteristics (Figure 5d). The bare CMUT devices exhibited a resonant frequency of 10.38 MHz when biased at a DC voltage of 6.2 V. After coating the SWCNT/peptide layer, the resonant frequency decreased to 10.37 MHz under the same DC bias voltage due to the mass-loading effect. A similar decrease in the resonant frequencies was observed for the devices coated with the peptide and PSAA layers (Figure S1). These results suggest that the CMUT devices were successfully fabricated and coated with peptide, SWCNT/peptide, and PSAA.

### 3.2. Chemical Response of CMUT Sensors to VOCs

**Figure 6:**
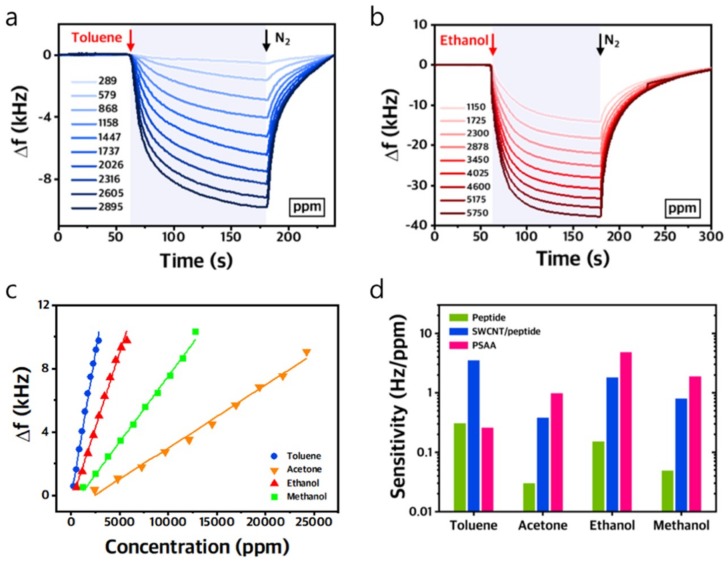
(**a**) Transient plot of the oscillation frequency of a CMUT sensor coated with the SWCNT/peptide layer in response to different concentrations of toluene. (**b**) Transient plot of the oscillation frequency of a CMUT sensor coated with the PSAA layer in response to different concentrations of ethanol. (**c**) Plot of maximum frequencies of the CMUT VOC sensor coated with SWCNT/peptide layer at various concentrations of four VOCs. (**d**) Plot of sensitivities of three CMUT sensors to toluene, acetone, ethanol, and methanol.

We measured the frequency response of three CMUT chemical sensors to four VOCs. First, the chamber that enclosed the CMUT devices was purged with nitrogen (N_2_) for 60 s. Different concentrations of the target analytes were then injected into the chamber for approximately 2 min, followed by a purge of nitrogen. The transient response of the CMUT sensors coated with the SWCNT/peptide layer and PSAA layer showed that frequency shifts increased as the concentrations of toluene and ethanol increased (Figure 6a,b). The maximum frequency shift at each concentration was plotted against the concentration of four VOCs to calculate the sensitivity (Figure 6c). The sensor exhibited a linear response to four VOCs including toluene, acetone, ethanol, and methanol. The SWCNT/peptide layer showed the highest sensitivity to toluene. We compared the responses of three CMUT sensors coated with the peptide, SWCNT/peptide, and PSAA layers to each gas (Figure 6d). The CMUT resonant sensors coated with SWCNT/peptide and peptide showed the highest sensitivity to toluene. Because SWCNT in the SWCNT/peptide layer increased the active surface area, the sensitivity of the CMUT sensor coated with the SWCNT/peptide layer to four VOCs were higher than that coated with the peptide alone. In contrast, the CMUT sensor coated with the PSAA layer showed the highest sensitivity to ethanol. Moreover, we also characterized the response time of our sensors by calculating the 10%–90% fall time and rise time (Figure S6). Both the fall and recovery time decreased as the sensors were exposed to a higher concentration of analytes. These results obtained at ambient temperature indicate that three CMUT devices exhibited different response patterns to all four VOCs which is sufficiently different for pattern recognition and multiplex detection of various VOCs. Although repeatability of the sensor response was not measured, we quantified the frequency stability of the oscillator read-out circuit interfaced with the CMUT sensor using Allan deviation. We observed a minimum Allan deviation of 2.2 Hz for gate time between 0.04 and 0.2 s.

**Figure 7:**
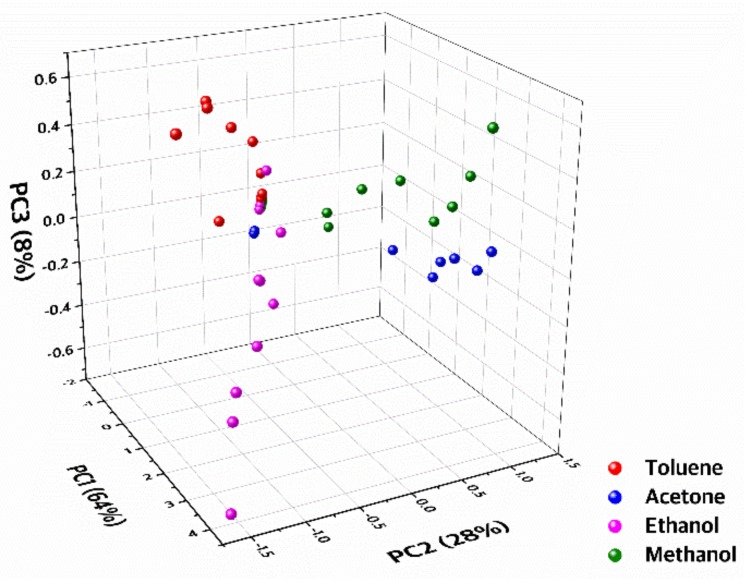
3D PCA plot of a data set composed of maximum frequencies and fall and rise times of three CMUT sensors coated with different chemically sensitive layers in response to various concentrations of four VOCs.

We analyzed the chemical experimental results of three CMUT sensors using a pattern recognition method to achieve selective detection (Figure 7). Principal component analysis (PCA), which is a statistical procedure to reduce the data complexity from a higher dimension to a lower dimension with high data correlation, was utilized as our pattern recognition method. Input data was composed of (1) maximum frequency shift values of three CMUT devices corresponding to each VOC gas with fixed concentration and (2) 10%–90% fall and rise time calculated from the transient responses (Figure S6). A total of 59 inputs was used. PCA results showed clear separation of VOC clusters which indicates selective detection of VOC gases. It was difficult to differentiate the input gas among four VOCs using a single sensor with a single chemically sensitive layer due to cross-sensitivity (Figure 6d). In contrast, using three sensors with the simultaneous multiplex detection scheme, we obtained extra fingerprints at a given instance of the same VOC exposure. Thus, the estimation accuracy of the gas concentration has improved by mapping the sensor output to the PCA results (Figure 7).

### 3.3. Portable CMUT Chemical Sensor with Wireless Transmission

**Figure 8:**
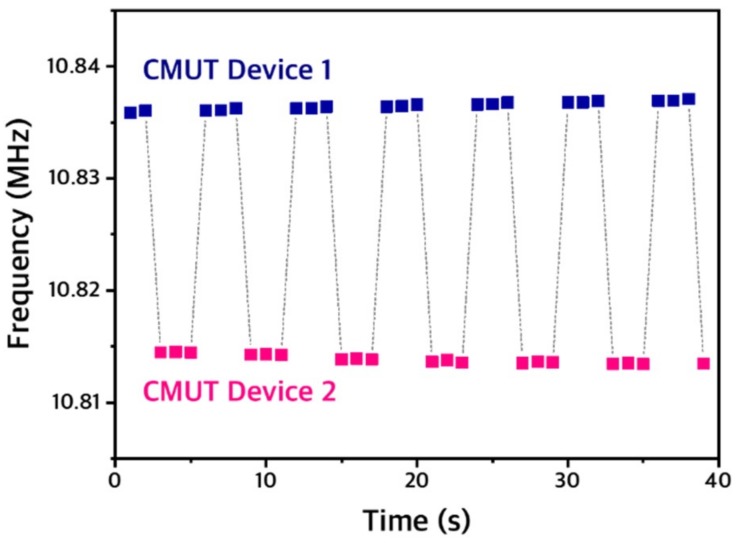
Transient plot of the output frequency of the single oscillator interfaced with two CMUT sensors through a relay, which switched between two sensors every 3 s.

The key idea behind our portable VOC sensor interface is to reduce the size of the sensor interface by using a single read-out circuit for multiple sensors (Figure 1). However, because an oscillator circuit is extremely sensitive to the impedance of a resonator and is subject to a finite start-up time, it is challenging to reliably start up an oscillation at every switching instance. To minimize the parasitic impedance imposed by the switch stage, we used a relay instead of an electrical switch. To confirm the correct operation of our switching scheme, the output frequency of a single oscillator interfaced to two CMUT sensors through a relay was measured over time. The relay switched between the two sensors every 3 s, which was controlled through the relay controller unit in MCU. The output of the single oscillator correctly showed the resonant frequencies of two CMUT sensors as the relay switched between the two sensors (Figure 8). Moreover, no significant drift was observed during the 3-s measurement period of each channel, which implies that the warm-up time for the oscillation is negligible in this system. Thus, the output of the single oscillator interfaced to multiple CMUT sensors through a relay can be reliably used for chemical detection.

**Figure 9:**
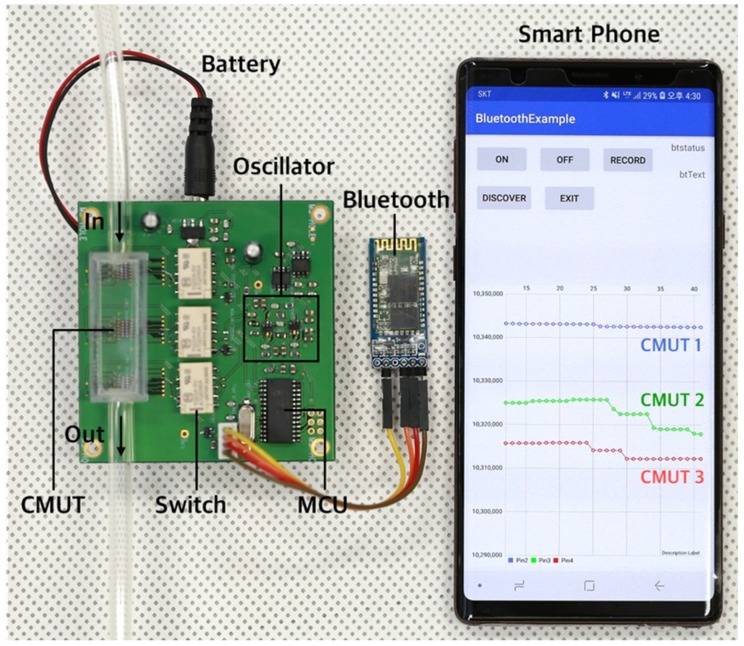
Photograph of the portable VOC sensor system composed of multiple CMUT sensors with multiplex detection scheme using a single read-out circuit and an electrically controlled relay. Oscillation frequencies of three CMUT sensors were simultaneously detected, transmitted, and displayed in the custom-designed Android application.

Finally, we implemented a compact portable system with wireless transmission and demonstrated a successful transmission of frequency shifts of three CMUT sensors when exposed to a VOC gas in real time (Figure 9). Upon exposure to toluene gas of 2895 ppm, the frequency shifts of three CMUT sensors coated with peptide, SWCNT/peptide, and PSAA layers were successfully measured using the single oscillator and the 3-s relay controlled through the MCU. The 3-s sampled 3-channel data was then transmitted to the smartphone and was displayed in the Android application in real time. Frequency shifts of each device were consistent with that observed in the bulk system. Thus, we successfully demonstrated real-time monitoring of VOCs using multiple CMUT sensors and a miniaturized single read-out interface.

## 4. Conclusions

We demonstrated a miniaturized CMUT-based VOC sensor system with multiplex detection scheme which achieves both selectivity and portability. We used multiple sensors that operate in ambient condition to obtain a larger number of fingerprints for pattern recognition but interfaced these sensors with a single read-out circuit to minimize the footprint of the overall system. By using Bluetooth transmission, this portable miniaturized sensor system with multiple sensors also transmit the data to a smartphone. In this work, we demonstrated the potential of a CMUT chemical sensor system as a portable sensing platform for real-time monitoring of VOCs.

## Supplementary Materials

The following are available online at https://www.mdpi.com/1424-8220/19/6/1401/s1, Figure S1: Plot of input impedance of CMUT device before/after the functionalization with peptide and PSAA layer, Figure S2: Transient plot of the oscillation frequency of a CMUT-based VOC sensor functionalized with SWCNT/peptide layer in response to varying concentrations of VOCs, Figure S3: Transient plot of the oscillation frequency of a CMUT-based VOC sensor functionalized with peptide layer in response to varying concentrations of VOCs, Figure S4: Transient plot of the oscillation frequency of a CMUT-based VOC sensor functionalized with PSAA layer in response to varying concentrations of VOCs, Figure S5: Plot of maximum frequencies of CMUT-based VOC sensors functionalized with peptide and PSAA layers at various concentrations of VOCs. Figure S6. Plot of (a) fall time and (b) rise (or recovery) time calculated from the transient measurements of two sensors (coated with colloid layer and PSAA layer) in response to various concentrations of toluene and ethanol.
