# Supplementary material for: A Capacitive Micromachined Ultrasonic Transducer-Based Resonant Sensor Array for Portable Volatile Organic Compound Detection with Wireless Systems
Source: Sensors (Basel). 2019 Mar 21;19(6):1401. doi: 10.3390/s19061401 (PMC6470568; doi:10.3390/s19061401)
Supplement: Supplementary file 1 [file sensors-19-01401-s001.pdf]

Supporting Information

A Capacitive Micromachined Ultrasonic Transducer-based Resonant Sensor Array for Portable Volatile Organic Compound Detection with Wireless System

Inug Yoon[1], Gayoung Eom[1,2], Sungwoo Lee[1], Bo Kyeong Kim[3], Sang Kyung Kim[3], and Hyunjoo J. Lee[1,*]

[1] School of Electrical Engineering, Korea Advanced Institute of Science and Technology (KAIST), Daejeon 34141, Republic of Korea; inug.yoon@kaist.ac.kr (I.Y.); gayoung.eom@kaist.ac.kr (G.E.); lsw9403@kaist.ac.kr (S.L.);

[2] Information & Electronics Research Institute, Korea Advanced Institute of Science and Technology (KAIST), Daejeon 34141, Republic of Korea

[3] Molecular Recognition Research Center, Korea Institute of Science and Technology (KIST), Seoul 02792, Republic of Korea; bk0357@khu.ac.kr (B.K.K.); sangk@kist.re.kr (S.K.K.)

* Correspondence: hyunjoo.lee@kaist.ac.kr; Tel.: +82-42-350-7436

Figure S1

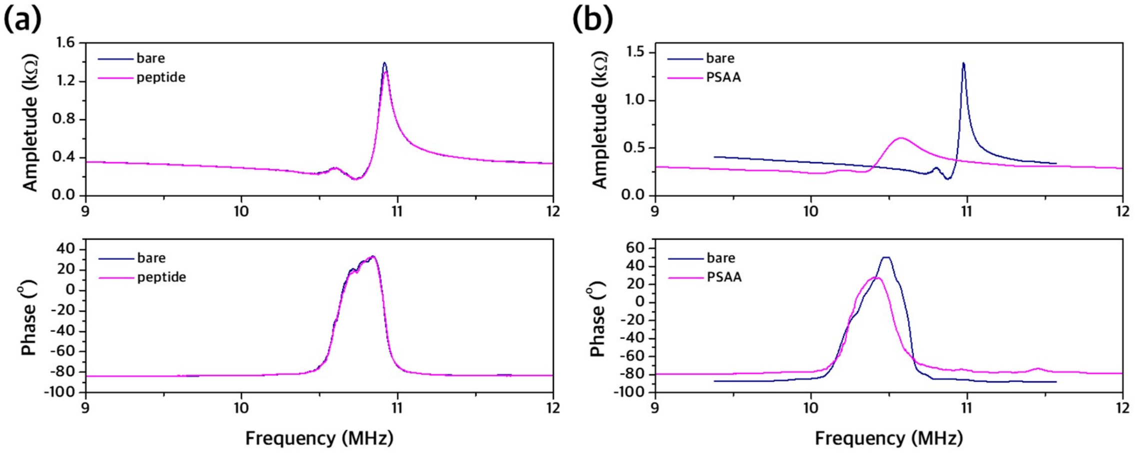

Figure S1. Plot of input impedance (amplitude and phase) of CMUT device before/after the functionalization with (a) peptide and (b) PSAA layer

Figure S2

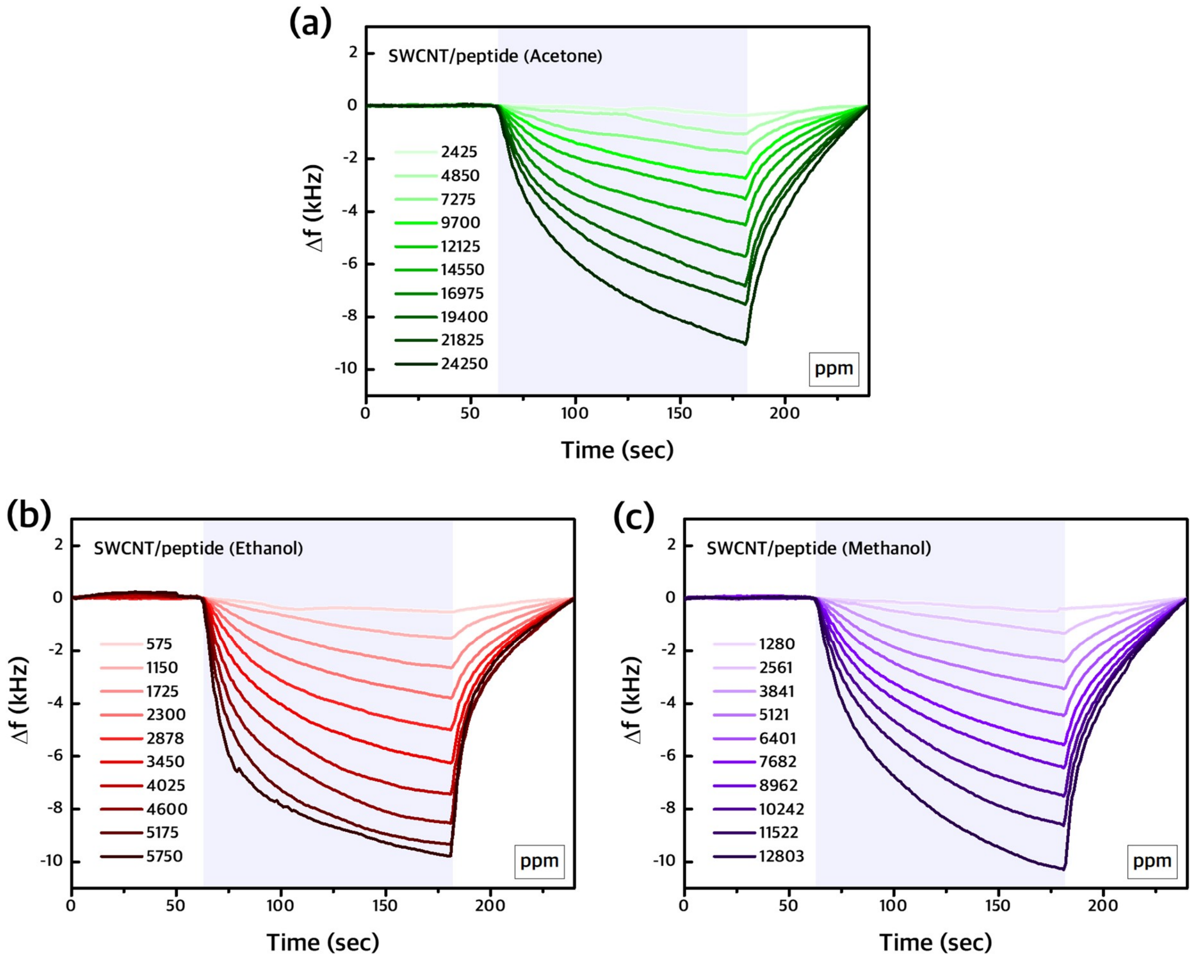

Figure S2. Transient plot of the oscillation frequency of a CMUT-based VOC sensor functionalized with SWCNT/peptide layer in response to varying concentrations of (a) acetone, (b) ethanol and (c) methanol.

Figure S3.

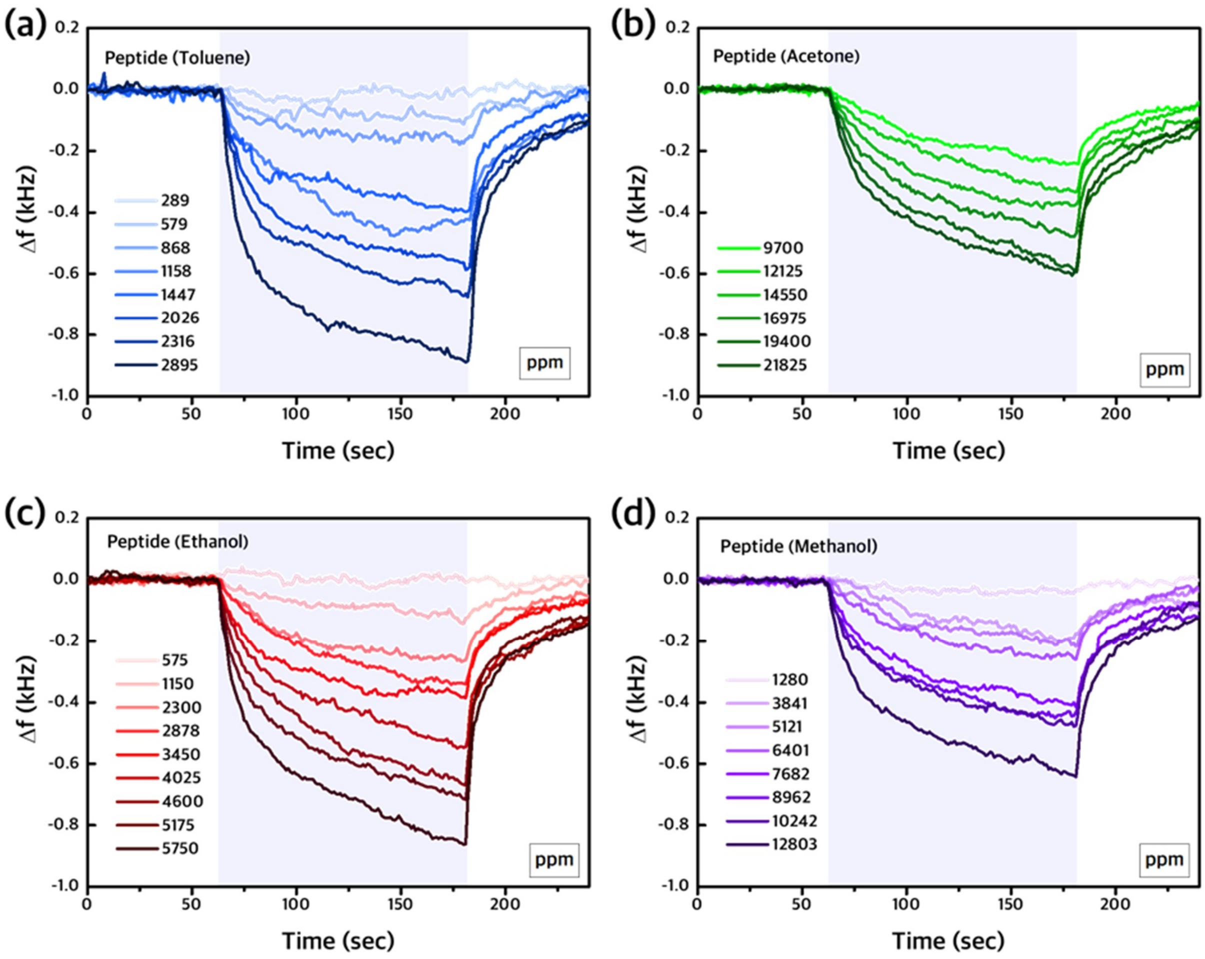

Figure S3. Transient plot of the oscillation frequency of a CMUT-based VOC sensor functionalized with peptide layer in response to varying concentrations of (a) toluene, (b) acetone, (c) ethanol and (d) methanol.

Figure S4.

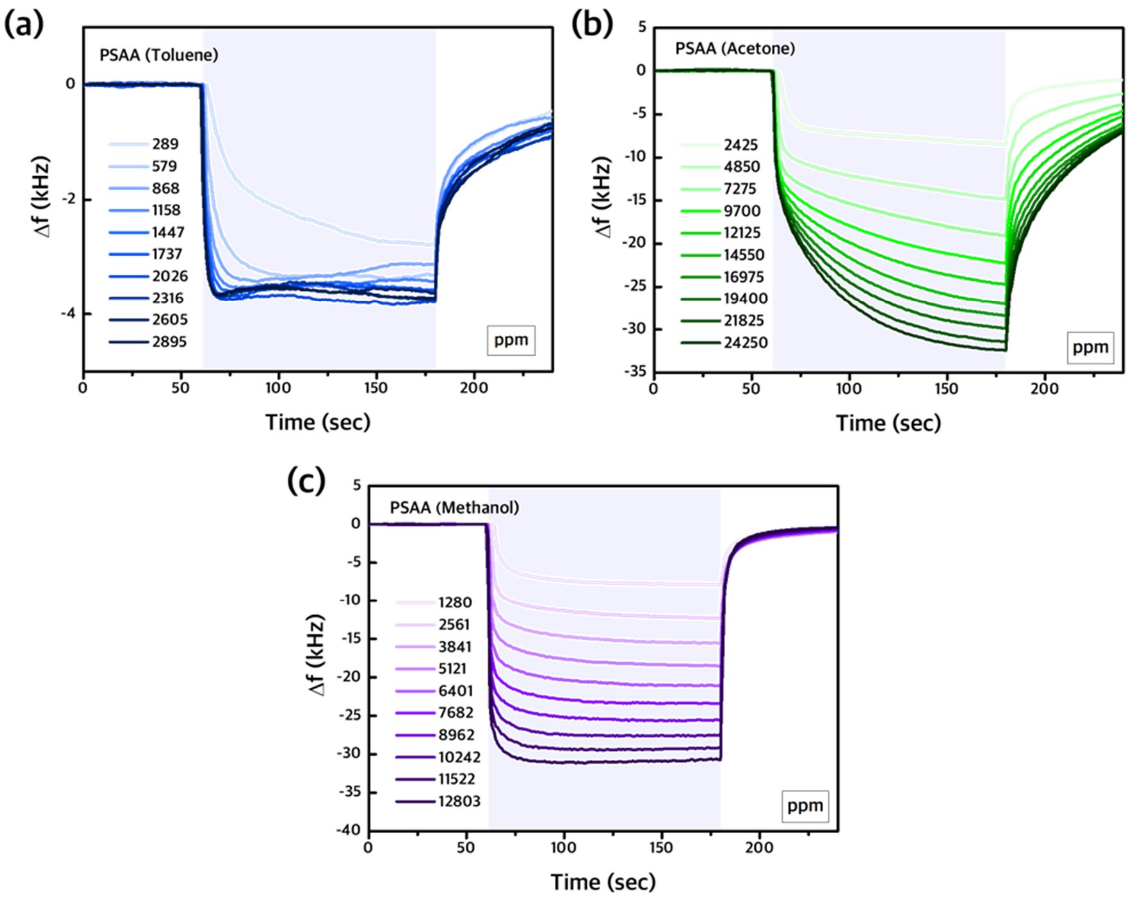

Figure S4. Transient plot of the oscillation frequency of a CMUT-based VOC sensor functionalized with peptide layer in response to varying concentrations of (a) toluene, (b) acetone, and (c) methanol.

Figure S5.

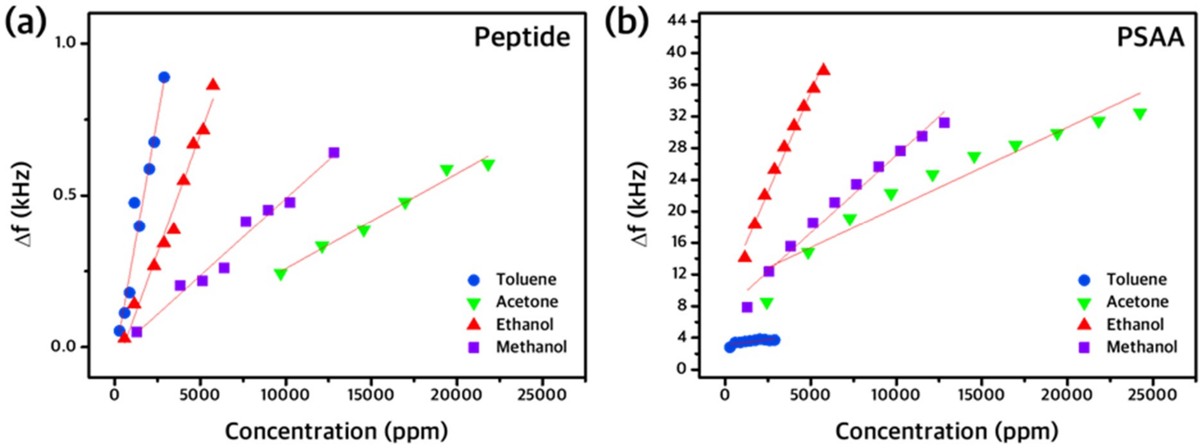

Figure S5. Plot of maximum frequencies of CMUT-based VOC sensors functionalized with (a) peptide and (b) PSAA layers at various concentrations of different VOCs.

Figure S6.

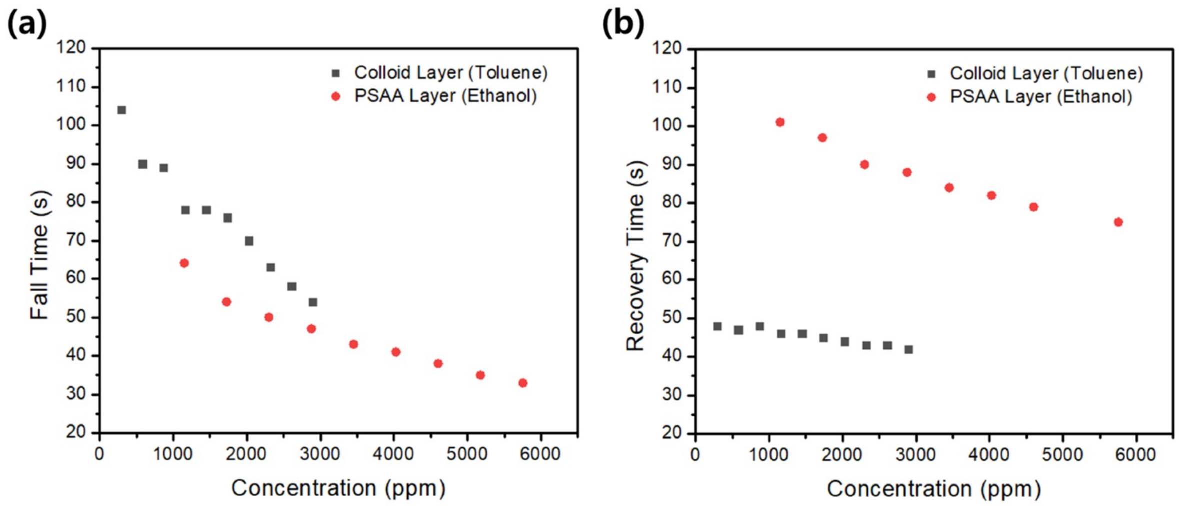

Figure S6. Plot of (a) fall time and (b) rise (or recovery) time calculated from the transient measurements of two sensors (coated with colloid layer and PSAA layer) in response to various concentrations of toluene and ethanol.
